# Development and Validation of a Stability-Indicating RP-HPLC Method for Edaravone Quantification

**DOI:** 10.3390/molecules30132866

**Published:** 2025-07-05

**Authors:** Riuna O’Neill, Okhee Yoo, Philip Burcham, Minh Nguyen, Lee Yong Lim

**Affiliations:** 1 Division of Pharmacy, School of Allied Health, University of Western Australia, Perth, WA 6009, Australia; riuna.oneill@research.uwa.edu.au (R.O.); okhee.yoo@uwa.edu.au (O.Y.); philip.burcham@uwa.edu.au (P.B.); minh.nguyen@uwa.edu.au (M.N.); 2 Institute for Paediatric Perioperative Excellence, University of Western Australia, Perth, WA 6009, Australia; 3 Wesfarmers Centre of Vaccines and Infectious Diseases, Telethon Kids Institute, University of Western Australia, Perth, WA 6009, Australia; 4 Centre for Optimisation of Medicines, School of Allied Health, University of Western Australia, Perth, WA 6009, Australia; 5 Division of Pharmacology and Toxicology, School of Biomedical Science, University of Western Australia, Perth, WA 6009, Australia

**Keywords:** edaravone, HPLC, motor neurone disease, assay, stability

## Abstract

Edaravone is used to treat motor neurone disease (MND) by slowing disease progression and prolonging survival time. Currently, it is available as an IV infusion (Radicava^®^, Jersey City, NJ, USA) and an oral liquid suspension (Radicava ORS^®^, Jersey City, NJ, USA). Development of novel edaravone formulations is still an active field of research that requires a validated stability-indicating assay capable of providing specific, precise, and accurate quantification of edaravone content. In this study, we developed and validated a stability-indicating reversed-phase high-performance liquid chromatography (RP-HPLC) method for edaravone quantification. Ten RP-HPLC methods based on the previously published literature were evaluated during method development. The optimal method employed a gradient method on an Agilent ZORBAX Extend-C18 column (150 × 4.6 mm, 5 µm) and produced a sharp and symmetrical drug peak. The method was further validated according to ICH Q2(R2) guidelines for specificity, linearity, sensitivity, accuracy, and precision. Successful separation of edaravone from void signals and degradant products was achieved. The method was precise and accurate at the concentration range of 6.8–68.6 µg/mL and was recommended to use without methyl hydroxybenzoate (MHB) as an internal standard.

## 1. Introduction

Edaravone is an amphiphilic, 2-pyrazolin-5-one derivative that was initially approved in 2001 as a treatment for acute ischaemic stroke due to its ability to mitigate the oxidative damage following a stroke event [1]. In 2015, edaravone received approval for the treatment of motor neurone disease (MND), as oxidative damage is believed to cause the progressive degeneration of motor neurones in the brain and spinal cord of MND patients [2]. Edaravone is able to slow the progression of MND and prolong survival time [3]. When first approved for the treatment of MND, edaravone was available only as an IV infusion (Radicava^®^). Patients must either attend a hospital or have carers make home visits to be administered with Radicava^®^. Dosing occurs daily for the first 14 days, and then daily for 10 out of 14 days for all subsequent dosing cycles, with 2-week drug-free periods between each cycle [4]. This treatment regimen is highly inconvenient and expensive. To address the limitations of Radicava^®^, an oral liquid suspension (Radicava ORS^®^) was developed and approved in 2022 [5,6]. Radicava ORS^®^, however, has other issues, such as poor bioavailability, poor storage stability, and inconvenient administration [4]. Thus, improving edaravone administration by creating novel edaravone formulations remains an active field of research.

When developing a novel edaravone formulation, it is crucial to have a validated assay capable of providing specific, precise, and accurate quantification of the edaravone content in the formulation. Additionally, as edaravone is a relatively unstable drug [7], it is essential that the validated assay is stability-indicating, i.e., capable of adequately separating the elution of edaravone and its degradation products, to allow for the monitoring of edaravone’s integrity during the manufacturing process and the determination of the shelf life and storage conditions of the optimised edaravone formulation.

Several research groups have published methods for edaravone quantification using methods such as high-performance liquid chromatography (HPLC) [8,9,10,11,12,13], ultraviolet (UV) spectrophotometry [8], liquid chromatography with tandem mass spectrometry (LC-MS/MS) [14], and thin-layer chromatography (TLC)–densitometry [15]. Of these, the HPLC technique is often preferred for drug assays as it is a relatively cost-effective and accessible method that offers precise, sensitive, and reproducible quantification of a wide range of drugs. Additionally, HPLC is the recommended method for edaravone assay in the Japanese Pharmacopoeia (JP) [12].

Several groups have published HPLC methods for edaravone quantification. Table 1 describes the equipment, chromatographic conditions, and, if provided, the validation results for these published HPLC methods [8,9,10,11,12,13]. All six methods employed columns with particle sizes of 5 µm, internal diameters of 4.6 mm, and lengths of 250 mm, except for the JP method, which recommends a shorter column length of 150 mm. All columns were C-18 columns apart from the one used by Tanaka et al. [9], who employed a column with adamantyl functional groups instead of octadecyl groups [16]. A variety of mobile phases were used, with isocratic elution commonly employed, aside from Baghel & Rajput [11], who used a gradient elution method. The injection volume, flow rate, detection wavelength, and temperature ranges were 10–20 µL, 0.5–1 mL/min, 240–295 nm, and ambient to 40 °C, respectively, with injection volume of 20 µL, flow rate of 1 mL/min, detection at 240 nm, and analysis at ambient temperature being the more common operation parameters. Not all groups validated the published methods according to the International Council for Harmonisation of Technical Requirements for Registration of Pharmaceuticals for Human Use (ICH Q2(R2)) guidelines [17]. Only Fanse and Rajput [8] and Baghel and Rajput [11] explicitly stated that their methods were validated according to ICH Q2 (R1) guidelines, while Tanaka et al. [9] did not report validation results at all, and Zeng et al. [10] provided incomplete datasets for the detection and quantification limits (DL and QL, respectively) and for the accuracy, specificity, and precision of their method. Of the three groups that provided comprehensive validation data [8], Patel et al. [13] reported the lowest intraday RSD (0.032–0.049%, *n* = 9) and interday RSD (0.086–0.094%, *n* = 9), with excellent drug recovery (100.01–100.19%, *n* = 9); however, their method had the largest DL and QL values (0.88 and 2.66 µg/mL, respectively). Conversely, Fanse & Rajput [8] reported the smallest DL and QL values (0.36 and 1.08 µg/mL, respectively). All three groups also demonstrated that their methods were specific for edaravone in the presence of formulation excipients, drug degradants, co-administered argatroban, and combinations thereof, with Baghel & Rajput [11] and Patel et al. [13] reporting that their methods were stability-indicating. None of the published methods utilised an internal standard (IS).

The objective of this study was to develop and validate a stability-indicating HPLC method to quantify edaravone. In the development of the edaravone assay, we have found the adoption of operation parameters reported for the published methods did not provide an optimal method for our purpose. This report outlines the steps taken to obtain the optimised conditions for the HPLC assay, followed by validation of the optimised assay using the ICH Q2(R2) guidelines.

## 2. Results and Discussion

### 2.1. HPLC Method Development

A total of 10 HPLC methods for edaravone quantification were trialled using mobile phases similar to those of the published HPLC methods for edaravone as described in Section 3. The results obtained for each method are summarised in Table 2 together with representative chromatograms. The methods are listed in chronological order, with reasons for rejection and subsequent changes made to improve results also described in Table 2.

The HPLC assay for edaravone was first developed (Method 1) using the exact mobile phase described by Baghel & Rajput [11]. The stationary phase used for Methods 1–8 (Agilent ZORBAX Extend C18 column, 250 × 4.6 mm, 5 µm) was selected for its similarity to those described in the published HPLC methods in Table 1 [8,9,10,11,12,13]. All other conditions for Method 1 were selected based on the published HPLC methods for edaravone quantification [8,10,11,13]. Method 1 did not yield a discernible drug peak, so the exact mobile phase described by Fanse & Rajput [8] was used for Method 2, which yielded a wide drug peak. A sharper peak is preferable to increase the specificity and accuracy of the method. Further adjustments to the mobile phase (Methods 3 and 4) did not improve the chromatograms (Table 2).

Methods 5, 6, and 7 applied the mobile phases respectively described by Tanaka et al. [9], JP [12], and Patel et al. [13]. Additionally, the edaravone calibration standards were prepared in MeOH instead of ACN to be in line with the compositions of the new mobile phases. Methods 5 and 7 both produced sharp drug peaks; however, method 7 was preferred as it yielded a symmetrical drug peak with narrower width of 1.2 min, and it utilised a salt-free mobile phase (35/65 (*v*/*v*) formic acid (0.1% in water)/50:50 ACN:MeOH). Methods 8 and 9 are adaptions of Method 7 and differ in the ratio of organic and aqueous components in the mobile phase. Decreasing the percentage of organic component in the mobile phase was aimed at better resolving the edaravone peak; however, it concurrently caused widening of the drug peak. To counter this, the injection volume was reduced from 20 µL to 5 µL, and a shorter column (Agilent Extend C18 column, 150 × 4.6 mm, 5 µm) was used in Methods 9 and 10.

**Figure 1:**
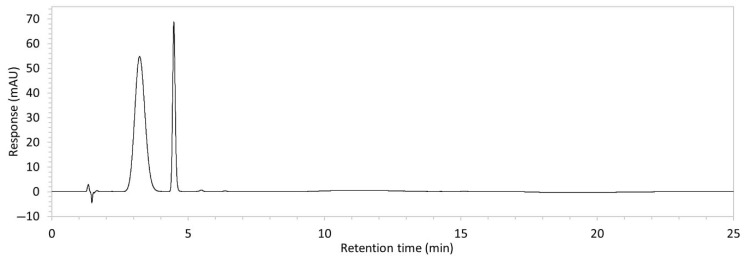
Chromatogram of a 90.9 µg/mL sample of edaravone with 18.2 µg/mL of internal standard (IS = methyl hydroxybenzoate) in MeOH obtained with optimised HPLC conditions (Method 10). IS produced a peak with AUC of 472.6 at a retention time (RT) of 4.5 min. Edaravone produced a peak with AUC 1475.3, width of 1.5 min, and symmetry value of 0.88 at RT of 3.2 min. Other smaller peaks (at RT 1.3 to 1.7, 5.5, and 6.3) were present in the blank sample (no edaravone).

**Figure 2:**
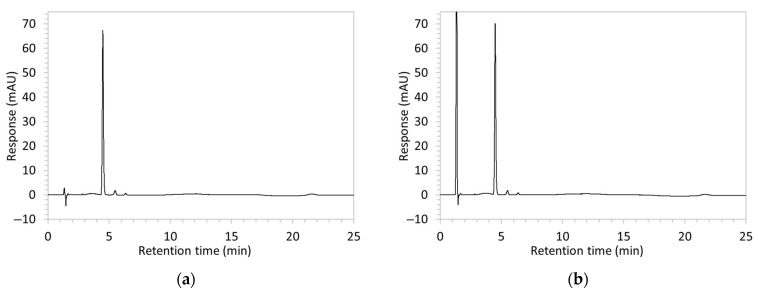

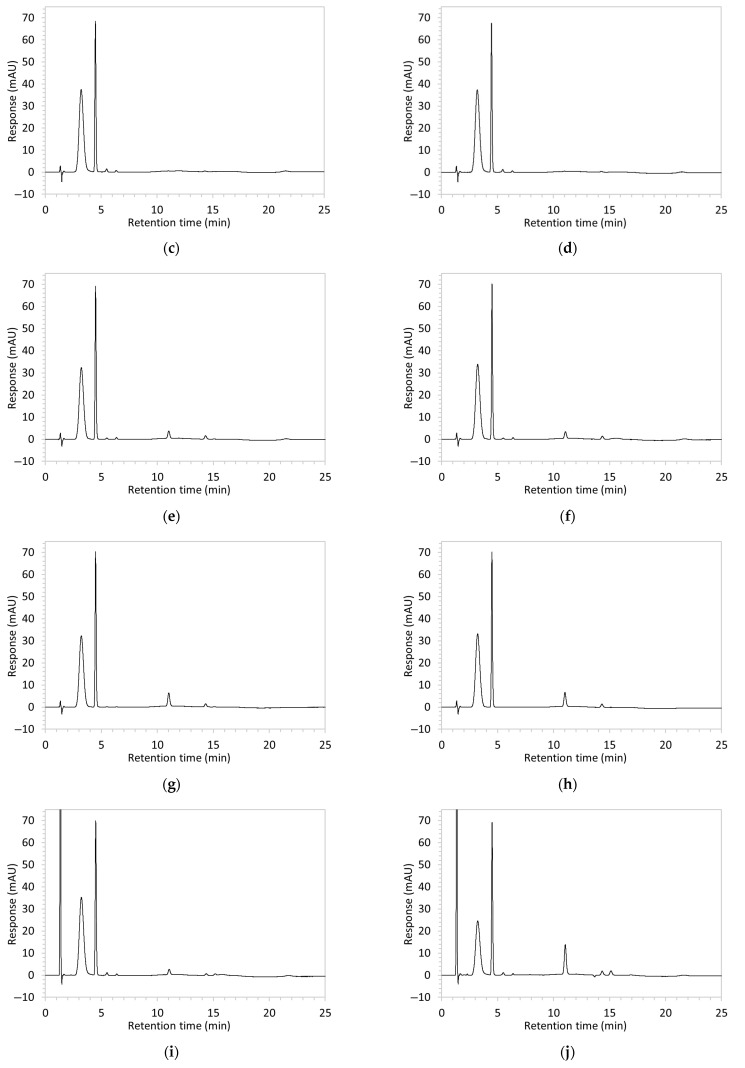
Chromatograms obtained with optimised HPLC conditions following exposure of placebo samples to forced degradation conditions in (**a**) water and (**b**) hydrogen peroxide (6%) and 60.6 µg/mL edaravone samples to forced degradation conditions in (**c**) water, (**d**) water with heating (70 °C for 45 min), (**e**) acid (0.1 M HCl), (**f**) acid with heating, (**g**) base (0.1 M NaOH), (**h**) base with heating, (**i**) hydrogen peroxide, or (**j**) hydrogen peroxide with heating. All samples were spiked with 18.2 µg/mL of IS prior to analysis. Edaravone and IS produced peaks at RT 3.2 and 4.5 min, respectively. Degradant peaks are present at RT 2.2, 11.0, 13.9, 14.4, and 15.6 min. Other peaks were produced in both placebo and edaravone samples at RT 1.2–1.8, 5.5–6.4, and 21.5–21.7 min. All placebo sample peaks are similar in size and shape, except for a peak at RT 1.2 min, which was much larger in the peroxide-exposed samples than in any others.

To ensure all degradants eluted within the runtime, a gradient elution method comprising three phases and two solvents (A: 0.1% formic acid in water; B: 50:50 ACN:MeOH) was adopted for Method 10. A lower percentage of B was used in the first phase with the aim to separate edaravone from void signals by slowing the elution of edaravone. The percentage of B was ramped up in the second phase to facilitate the elution of degradant peaks within the runtime before the percentage of B was returned to the initial level in the third phase. This approach for Method 10 was successful at producing an acceptable chromatogram (Figure 1), where a 90.9 µg/mL sample of edaravone produced a sharp peak with AUC 1475.3 and a symmetry value of 0.88 at an RT of 3.2 min. The edaravone peak was well separated from the IS peak at 4.5 min RT. Importantly, the edaravone peak was also completely separated from the peaks of the edaravone degradant products (Figure 2), which all eluted within the 25 min runtime. Thus, Method 10 was deemed to be the optimised method and was further validated according to the ICH guidelines.

### 2.2. Specificity

Chromatograms of edaravone and placebo samples following forced degradation with water, acid, base, peroxide, and/or heat are provided in Figure 2, and Table 3 lists the RT and AUC of the peaks for edaravone, IS, and degradant products. Peaks attributable to edaravone degradation products were those identified as present in the degradation samples but absent in the blank samples, whereas peaks observed at RT 1.2–1.8, 5.5–6.4, and 21.5–21.7 min that were present in both the degraded samples and blank sample were attributed to noise (Figure 2a,b). For all samples, the edaravone peak was observed at RT 3.2 min, but it had different AUCs, indicating varying extents of degradation in the different solvent systems. Edaravone exposed to water at room temperature showed a relatively intact edaravone peak and one small degradant peak at RT 14.4 min (Figure 2c), with heating (70 °C for 45 min) enlarging the peak at RT 14.4 min (AUC 5.4) and introducing another peak (AUC 1.6) at RT 11.0 min (Figure 2d). The degradant peaks at RT 11.0 and 14.4 min were also observed in edaravone samples exposed to acid, base, and peroxide, with and without heat. Samples degraded in peroxide, with or without heating, also showed additional degradant peaks at RT 2.2 and 15.6 min, with a further peak observed at 13.9 min for the peroxide sample exposed to heat. The AUCs for the degradant peaks were highest in the sample exposed to heating and peroxide, which also had the smallest edaravone peaks, suggesting that edaravone degraded to the highest extent in this sample. The results are in agreement with published data, as edaravone is known to exhibit strong radical scavenging activity by donating an electron to reactive oxygen species and undergoing a series of reactions to form new compounds, including, but not limited to, edaravone trimers [18,19]. Under the photolytic degradation conditions employed in this study, the edaravone peak remained unchanged, and no degradant peaks were formed, which suggests that edaravone did not experience degradation from 24 h of UV light exposure.

All degradant and noise peaks were well separated from the edaravone peak at RT 3.2 min for all samples analysed. Therefore, this method is regarded as being specific and stability-indicating for edaravone.

### 2.3. Linearity and Sensitivity

Linear calibration curves were obtained for the edaravone standard solutions, determined with and without IS (R^2^ > 0.99) (Table 4 and Table 5). The DL and QL determined from the calibration curves were both found to be similar when determined without the IS (0.340 and 1.03 µg/mL, respectively; Table 4) or with the IS (0.352 and 1.07 µg/mL, respectively; Table 5). This suggests that the method’s sensitivity was not improved with IS inclusion. All DL and QL values were lower than those obtained by Patel et al. [13] (0.88 and 2.66 µg/mL, respectively), who used a similar mobile phase but higher edaravone concentration range for the calibration curve (120–360 vs. 1.8–90.9 µg/mL), and Baghel and Rajput [11] (0.5341 and 1.6186 µg/mL, respectively), who employed a gradient elution approach similar to this study. The DL and QL values were, however, comparable to those obtained by Fanse & Rajput [8] (0.3566 and 1.08 µg/mL, respectively), with a method that did not report specificity against degradant products of edaravone.

### 2.4. Precision and Accuracy

Precision and accuracy analyses are crucial to ensure that the measured edaravone concentrations are reliable and as close as possible to the true value. As the ICH Q2(R2) guidelines do not provide guidelines for acceptable precision, an RSD of <5% was chosen as the acceptable limit for this study [10]. The RSD (%) of intraday precision for edaravone remained below 1% except for the 1.8 µg/mL edaravone sample with the IS, which showed an RSD of 1.2% (Table 6). These results are comparable to the RSD values obtained by Patel et al. [13]. Compared with the intraday precision RSD values, the interday precision RSD values were higher, ranging from 1.0 to 3.9% (Table 7). However, all interday RSD values were still within the acceptance limit of <5%, indicating that the method was precise.

The ICH also does not give specific threshold values for accuracy, and an acceptance value of 95–105% drug recovery (with low SD values) was used for this study. Most recovery values for edaravone in this study met this criterion; however, the interday percent recovery readings for 113.6 µg/mL edaravone without the IS exceeded 105% (Table 7). This was not unexpected, as the highest edaravone concentration level was outside the calibration concentration range. Similarly, the intra- and interday percent recovery readings for 68.2 µg/mL and 113.6 µg/mL edaravone with the IS also exceeded 105% (Table 6 and Table 7).

Overall, the results indicated that the method was most accurate between 1.8 and 68.6 µg/mL edaravone without the IS and between 1.8 and 22.7 µg/mL with the IS. Thus, it is recommended that the assay be performed without the IS, as the IS does not improve precision and negatively impacts the accuracy and sensitivity of the method.

## 3. Materials and Methods

### 3.1. Materials

All chemicals were of analytical grade. Edaravone was purchased from Merck Life Science (Bayswater, Australia). MeOH was from Honeywell Burdick & Jackson (Muskegon, MI, USA). ACN was purchased from RCI Labscan (Bangkok, Thailand). Formic acid, acetic acid, solid NaOH pellets, and H_2_O_2_ (30%) were purchased from ChemSupply Australia (Gillman, Australia). Na_2_HPO_4_, ammonia solution, and ammonium acetate were purchased from Ajax Finechem (Taren Point, Australia). HCl (32%) was purchased from ACE Chemical Company (Camden Park, Australia). Water was deionised and filtered. Methyl hydroxybenzoate BP (MHB) was purchased from PharmAust Manufacturing (Malaga, Australia).

### 3.2. HPLC Method Development and Final Chromatographic Conditions

HPLC analysis was performed on an Agilent 1260 Infinity II HPLC system (Agilent Technologies Australia, Mulgrave, Australia) equipped with binary pump, autosampler, and diode array detector HS. All solutions for HPLC were filtered through a 0.45 µm nylon membrane (Merck Life Science, Bayswater, Australia). Chromatograms were generated and analysed using the Agilent OpenLab CDS software (version 2.4.0, Knauer, Berlin, Germany).

Multiple operating parameters, adapted from the published methods and described in Table 8, were used to develop a suitable HPLC assay for edaravone. Optimised chromatographic separations (Method 10, Table 8) were achieved at ambient temperature using an Agilent ZORBAX Extend-C18 column (150 × 4.6 mm, 5 µm; Agilent Technologies, Chatswood, NSW, Australia) with a gradient elution method as follows: (time (min)/%B) − 0/40, 7/40, 11/50, 16/50, 20/40, 25/40, wherein the mobile phase consisted of aqueous formic acid (0.1%; A), and 50:50 (v/v) ACN:MeOH (B). Analysis was performed over 25 min, at a flow rate of 1 mL/min, injection volume of 5 µL, and with MHB as the internal standard (IS).

### 3.3. Preparation of Calibration Standards for Optimised Method

Edaravone stock solution was prepared fresh each day by dissolving edaravone in methanol at 100 µg/mL. The IS stock solution was prepared by dissolving MHB in methanol at 200 µg/mL, and the solution was stored at −80 °C until use. To prepare the edaravone calibration standards, edaravone stock solution was diluted with methanol to 1 mL, and 100 µL of IS stock was added to provide final edaravone concentrations ranging from 1.8 to 90.9 µg/mL and IS concentration of 18.2 µg/mL. Samples used for methods 1–9 were prepared similarly but did not include IS stock, and they yielded final edaravone concentrations ranging from 2 to 100 µg/mL.

### 3.4. Validation Methodology

Validation of the optimised HPLC method was performed according to ICH Q2(R2) guidelines [17].

#### 3.4.1. Specificity

Specificity describes the extent to which the method is able to measure the analyte in the presence of other substances that may potentially interfere with the analysis of the target analyte [17]. Edaravone is known to rapidly undergo oxidation and is susceptible to alkaline hydrolysis [7]. To assess method specificity, edaravone samples were analysed after forced degradation in UV light, acid, base, peroxide, water, and/or heat. Specificity of the method was assured if the chromatogram for the degraded samples showed complete separation of the edaravone peak from those of its degraded products.

For acid, base, peroxide, water, and heat degradation, 1 mL samples containing 1 mg/mL edaravone in 0.1 M HCl, 0.1 M NaOH, 6% H_2_O_2_, or water were prepared in duplicates, with one sample maintained at ambient temperature for 45 min, while the other was heated at 70 °C for 45 min. Samples (1 mL) containing HCl, NaOH, and H_2_O_2_ were neutralised with 0.5 mL of NaOH, HCl, and MeOH, respectively. All prepared samples were stored at −20 °C until analysis. For photolytic degradation, a solution of 1 mg/mL edaravone in methanol was exposed to UV light for 24 h, and then was diluted 2:1 with methanol. To prepare the samples for analysis, the samples were thawed, then diluted ten-fold with a 50/50 mixture of water/MeOH, and then spiked with 100 µL of IS stock solution to yield final concentrations of 60.6 µg/mL edaravone and 18.2 µg/mL IS. Degradation samples used for Methods 1–9 were prepared similarly but did not include IS stock and yielded a final edaravone concentration of 66.7 µg/mL.

#### 3.4.2. Linearity and Sensitivity

Linearity of an analytical method refers to its ability to provide test results that are directly proportional to the concentration of an analyte in a sample within a specified range. Good linearity is crucial, as it would reversely allow one to quantify the analyte in a test sample. In this study, linearity was evaluated by constructing a calibration curve using 11 concentration levels of edaravone (0, 1.8, 3.6, 5.4, 7.2, 9.1, 18.2, 36.4, 54.5, 72.7, 90.9 µg/mL) with and without the IS. Linear regression analysis was performed to evaluate the correlation between the peak area under the curve (AUC) and edaravone concentration, and the correlation coefficient of the regression line was used to assess linearity (R^2^ > 0.99).

Sensitivity of an analytical method refers to its ability to detect and quantify low amounts of the analyte in a sample. Sensitivity is assessed through the detection limit (DL) and quantification limit (QL), which are the minimum concentrations of the analyte that can be reliably detected and quantified with acceptable accuracy and precision, respectively [17]. The calibration curves (*n* = 3) were used to determine the DL and QL of edaravone, using the following equations, where σ represents the standard deviation of the response and S represents the mean slope of the calibration curves:

$$DL=\frac{3.3\sigma }{S}$$

$$QL=\frac{10\sigma }{S}$$

#### 3.4.3. Precision and Accuracy

The accuracy of an analytical method is the closeness of agreement between the value determined by the method and the true or expected value expressed as the percent recovery of a known added amount of analyte in a sample. The precision of an analytical method is the closeness of agreement between a series of measurements obtained from multiple sampling of the same homogeneous sample under the prescribed conditions expressed as the relative standard deviation (%) of the found analyte concentrations. It is important to validate an analytical method for accuracy and precision to ensure that the method will consistently give a value for the quantity of an analyte in a sample that is close to the true amount.

The ICH Q2(R2) guidelines state that for both accuracy and precision assessments, a minimum of 9 determinations covering the specified range for the procedure should be used. For this study, precision and accuracy were determined using triplicate samples containing 1.8, 6.8, 22.7, 68.2, and 113.6 µg/mL edaravone. All samples also contained 18.2 µg/mL of IS. Triplicate samples were prepared similarly but using separate edaravone stock solutions made fresh on the day of analysis.

Precision was assessed under the same conditions within the same day (intraday precision) and across 3 days (interday precision), and calculating the RSD (%) of the predicted concentrations. Accuracy analysis was conducted by determining the drug recovery of the samples at each concentration level, expressed as follows:

$$Recovery\left(\%\right)=\frac{Measured\;concentration}{Nominal\;concentration}\times 100$$

## 4. Conclusions

The RP-HPLC method for the quantification of edaravone was developed. The method produced a sharp and symmetrical peak that was well separated from void signals present in the blank and from degradants produced following edaravone exposure to peroxide, acid, base, and heat. Validation was performed in accordance with ICH Q2(R2) guidelines, and the HPLC method was found to be precise and accurate at the concentration range of 6.8–68.6 µg/mL. This is an improvement upon most of the published HPLC methods listed in Table 1, wherein only the method by Baghel and Rajput [11] was both stability-indicating and validated according to ICH standards. None of the published methods attempted using an IS. In this study, MHB was selected as an IS, but it was found that the use of MHB as an IS did not improve edaravone quantification using the HPLC conditions, and in fact negatively impacted the accuracy of the method; therefore, it is not recommended to use MHB as an IS with the method. In conclusion, the developed method was successfully validated, was stability-indicating, and may be used for the quantification of edaravone in samples that may potentially contain edaravone degradation products.

## Figures and Tables

**Table 1:** Summary of some HPLC methods for edaravone quantification found in the published literature from 2010 to 2025.

Reference	Fanse & Rajput [8]	Tanaka et al. [9]	Zeng et al. [10]	Baghel & Rajput [11]	JP [12]	Patel et al. [13]
**Equipment**	Shimadzu LC system; Shimadzu LC-20AT pump, Shimadzu SPD-20AV UV detector, Rheodyne 7725 injector, Spinchrome software	Not reported	Shimadzu HPLC system	Waters Acquity; PDA UV detector, Empower-2 software	LC800 HPLC system	Shimadzu LC system; Shimadzu LC-10 single pump, Shimadzu SPD-10A UV detector, Winchrome software
**Stationary phase**	Kromasil C18 (250 × 4.6 mm, 5 µm)	CAPCELL PAK ADME (250 × 4.6 mm, 5 µm)	Hedera ODS C18 (250 × 4.6 mm, 5 µm)	Thermo Scientific BDS Hypersil RP-C18 (250 × 4.6 mm, 5 µm)	Inertsil ODS-3 C18 (150 × 4.6 mm, 5 µm)	Inert cell C18 (250 × 4.6 mm, 5 µm)
**Mobile phase**	Isocratic: 55% water, 45% ACN	Isocratic: 60% MeOH, 40% NaHPO_4_ (aqueous; 40 mM)	Isocratic: 50% MeOH, 50% KH_2_PO_4_ (aqueous; 0.05 M; pH 3.5)	Gradient (time (min)/%B): 0.01/05, 04/15, 10/25, 12/45, 14/25, 16/15, 17/05, 20/05 A: Ammonium acetate buffer (10 mM; pH 5.8) B: 90:10 ACN:MeOH	Isocratic: 50% MeOH, 50% Acetic acid (1% in water)	Isocratic: 35% formic acid (0.1% in water), 33% ACN, 32% MeOH
**Injection volume (µL)**	20	Not reported	20	20	10	20
**Flow rate (mL/min)**	1	0.5	1	0.8	0.95	1
**Detection wavelength (nm)**	243	295	240	244	240	260
**Temperature (°C)**	Ambient	Not reported	35	34	40	Ambient
**Description of peak**	Chromatogram showed a sharp and symmetrical peak	Chromatogram not provided	Chromatogram not provided	Chromatogram showed a sharp and symmetrical peak	Chromatogram showed a sharp and symmetrical peak	Chromatogram showed a sharp and symmetrical peak
**Edaravone RT (min)**	4.25	Not reported	8.5	Approx. 11.6, estimated from published chromatogram	Approx. 4, estimated from published chromatogram	4.13
**Linearity concentration range (µg/mL)**	10–80	Not reported	10.15–101.5	10–300	Not reported	120–360
**DL (µg/mL)**	0.3566	Not reported	Not reported	0.5341	Not reported	0.88
**QL (µg/mL)**	1.08	Not reported	Not reported	1.6186	Not reported	2.66
**Precision (RSD)**	Intraday: 0.1378% (*n* = 3) Interday: 1.0239% (*n* = 9)	Not reported	> 5% RSD	Intraday: 0.2011% Interday: 0.3035%	Not reported	Intraday: 0.032–0.049% (*n* = 9) Interday: 0.086–0.094% (*n* = 9)
**Accuracy (% drug recovery)**	99–101% (*n* = 9)	Not reported	Not reported	> 99%	Not reported	100.01–100.19% (*n* = 9)
**Specificity**	Complete separation of the drug peak from peaks of unnamed excipients	Not reported	Not reported	Complete separation of the drug peak from its degradation products	Not reported	Complete separation of the drug peak from its degradation products and from argatroban
**Stability indicating?**	Not reported	Not reported	Not reported	Yes; the drug was subjected to acid, base, oxidative, thermal, photolytic, and high-humidity degradation conditions	Not reported	Yes; the drug was subjected to acid, base, oxidative, hydrolytic, thermal, and photolytic degradation conditions

JP: Japanese pharmacopoeia; ACN: acetonitrile; MeOH: methanol; RT: retention time; DL: detection limit; QL: quantitation limit; RSD: relative standard deviation.

**Table 2:** Overview of stability-indicating HPLC method development for the analysis of edaravone. Chromatograms and reasons for excluding attempted HPLC methods, and the changes made to resolve the issues, are given. Method descriptions are given in Section 3.

**Method**	**Chromatogram**	**Results**	**Changes Made to Resolve Issues, Leading to Next Method**
1 *	100 µg/mL edaravone: 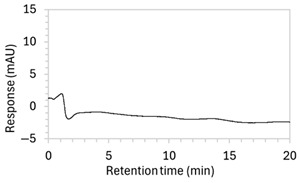	No apparent drug peak observed.	Mobile phase composition was modified.
2 *	100 µg/mL edaravone: 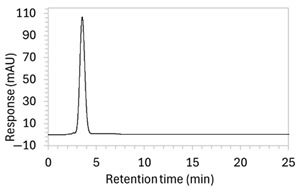	Drug peak observed, but peak was broad (1.9 min wide).	Increased the proportion of organic solvent in mobile phase to facilitate faster elution of edaravone.
3	100 µg/mL edaravone: 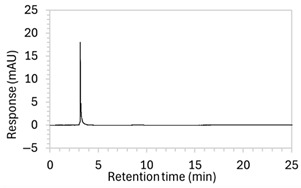	No drug peak observed; a new peak appeared at RT 3.1 min, also present in the blank.	Changed isocratic method to gradient elution to improve control over edaravone elution.
4	100 µg/mL edaravone: 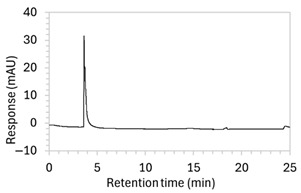	No drug peak observed; peak present at RT 3.7 was also present in the blank.	Modified both mobile phase and the sample solvent. Reverted to isocratic elution.
5 *	100 µg/mL edaravone: 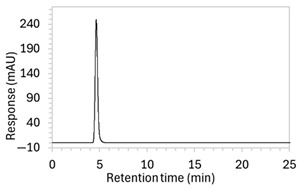	Drug peak detected but significant tailing resulted in increased peak width (2.1 min wide, including tail end).	Mobile phase composition was adjusted.
6 *	100 µg/mL edaravone: 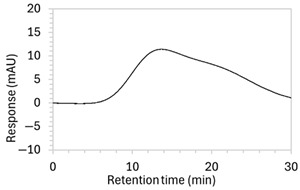	No drug peak observed.	Further modifications to the mobile phase.
7 *	100 µg/mL edaravone: 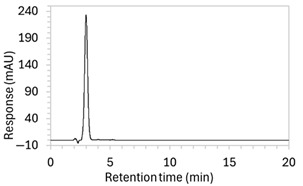	Drug peak was sharp and symmetrical (1.2 min wide) but eluted too early, resulting in poor resolution from void signal.	Decreased the organic solvent percentage in the mobile phase to slow edaravone elution. Injection volume was reduced to counteract peak broadening.
8	(a) 100 µg/mL edaravone: 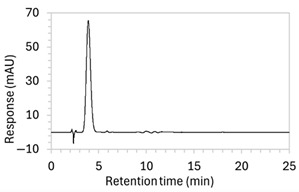 (b) 66.7 µg/mL edaravone after acid degradation: 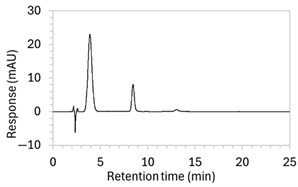	(a) Drug peak observed at 3.9 min, with improved separation from void signals. However, peak width increased to 1.8 min. (b) Drug degradant peaks at RT 8.5 and 13 min were adequately separated from the drug peak.	The percentage of organic solvent in the mobile phase was decreased to slow elution and enhance separation from void signals. To counteract peak broadening due to slower elution, a shorter HPLC column was used to moderate elution rate.
9	(a) 100 µg/mL edaravone: 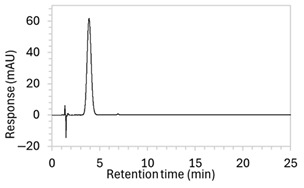 (b) 66.7 µg/mL edaravone after acid degradation: 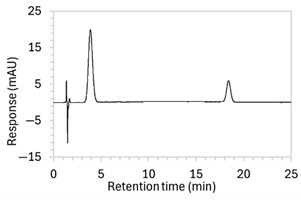	(a) Drug peak was well resolved from void signals, but remained 1.8 min wide. (b) The main degradant peak (RT 18.5 min) eluted much later than in the previous method, raising concerns that smaller degradant peaks may not have eluted within the runtime.	Changed to gradient elution method to retain separation between the drug and void signals while ensuring complete elution of all degradants. The detection wavelength was changed from 243 to 250 nm to enhance peak visibility. Internal standard was included.
10	See Figure 1 and Figure 2.	Optimised conditions achieved. Drug peak was sharp (1.5 min wide) with complete separation from all degradants.	-

* Method mobile phase is exactly as described by a method in the current published literature.

**Table 3:** Drug content (% of water-exposed edaravone sample), size (AUC), and RT of edaravone, IS, and degradant peaks detected in chromatograms of 60.6 µg/mL edaravone following forced degradation conditions as pictured in Figure 2.

	Edaravone Peak		IS (MHB) ^5^ Peak	Degradant Peaks				
Peak RT (min)	3.2		4.5	2.2	11.0	13.9	14.4	15.6
Forced Degradation Condition	Drug Content (% of Water-Exposed Edaravone)	Peak AUC						
Water	100	1055.5	468.4	-	-	-	3.9	-
Water + heating ^1^	99.1	1045.6	459.8	-	1.6	-	5.4	-
Acid ^2^	83.4	880.5	471.2	-	43.2	-	20.5	-
Acid + heating	87.8	926.7	482.1	-	39.5	-	18.6	-
Base ^3^	83.9	885.7	478.0	-	76.7	-	18.1	-
Base + heating	86.3	910.5	479.8	-	81.4	-	19.7	-
Peroxide ^4^	94.5	997.0	482.4	0.8	31.9	-	11.1	8.2
Peroxide + heating	67.0	707.2	476.4	4.6	170.1	5.5	24.5	27.6

^1^ heating at 70 °C for 45 min; ^2^ 0.1 M HCl; ^3^ 0.1 M NaOH; ^4^ 6% H_2_O_2_; ^5^ methyl hydroxybenzoate.

**Table 4:** Range, regression equation, coefficient of correlation (R), average slope, and standard deviation (SD) of y-intercepts of the edaravone calibration curves, and the DL and QL of the HPLC method, without the IS. Calibration curves were constructed using 11 different concentration levels of edaravone ranging from 0–90.9 µg/mL.

Run	Linearity Range (µg/mL)	Regression Equation	Coefficient of Correlation (R)	Slope (Average)	Y-Intercept (SD)	DL (µg/mL)	QL (µg/mL)
**1**	0–90.9	y = 15.6013x − 1.5570	0.999981	15.5859	1.6041	0.340	1.03
**2**	0–90.9	y = 15.7426x − 0.4545	0.999940				
**3**	0–90.9	y = 15.4136x − 1.6134	0.999988				

**Table 5:** Range, regression equation, coefficient of correlation (R), average slope, and standard deviation (SD) of y-intercepts of the edaravone calibration curves, and the DL and QL of the HPLC method, with the IS. Calibration curves were constructed using 11 different concentration levels of edaravone ranging from 0–90.9 µg/mL.

Run	Linearity Range (µg/mL)	Regression Equation	Coefficient of Correlation (R)	Slope (Average)	Y-Intercept (SD)	DL (µg/mL)	QL (µg/mL)
**1**	0–90.9	y = 0.0338x − 0.0022	0.999941	0.0333	0.0036	0.352	1.07
**2**	0–90.9	y = 0.0333x − 0.0012	0.999958				
**3**	0–90.9	y = 0.0328x − 0.0050	0.999988				

**Table 6:** Accuracy and intraday precision for the assays of edaravone samples at various nominal concentrations (*n* = 3).

Nominal Edaravone Concentration (µg/mL)	Measured Edaravone Concentrations (µg/mL)		Accuracy (Mean Measured Drug Recovery ± SD (%))		Precision (RSD (%))	
	Without IS	With IS	Without IS	With IS	Without IS	With IS
1.8	1.76 1.76 1.73	1.80 1.77 1.76	95.7 ± 0.8	97.2 ± 1.1	0.9	1.2
6.8	6.931 6.945 6.970	6.971 6.883 6.953	101.4 ± 0.3	101.2 ± 0.7	0.3	0.7
22.7	23.37 23.26 23.31	23.60 23.47 23.70	102.1 ± 0.2	103.3 ± 0.5	0.2	0.5
68.2	70.97 70.94 70.93	71.48 72.21 72.61	103.5 ± 0.0	105.2 ± 0.8	0.0	0.8
113.6	119.7 119.7 119.4	120.7 120.3 118.6	104.7 ± 0.1	105.0 ± 1.0	0.1	0.9

**Table 7:** Interday precision for the assays of edaravone samples at various nominal concentrations (*n* = 9).

Nominal Edaravone Concentration (µg/mL)	Measured Edaravone Concentrations (µg/mL)						Mean Measured Drug Recovery ± SD (%)		Precision (RSD (%))	
	Without IS			With IS			Without IS	With IS	Without IS	With IS
	Day 1	Day 2	Day 3	Day 1	Day 2	Day 3				
1.8	1.8 1.8 1.7	2.0 2.0 2.0	1.9 1.9 1.9	1.8 1.8 1.8	2.0 2.0 2.0	2.1 2.1 2.0	100.7 ± 3.9	101.7 ± 3.5	3.9	3.4
6.8	6.9 6.9 7.0	7.4 7.5 7.4	7.2 7.3 7.1	7.0 6.9 7.0	7.5 7.4 7.5	7.4 7.4 7.5	102.3 ± 1.1	102.8 ± 1.5	1.0	1.4
22.7	23.4 23.3 23.3	25.3 25.2 25.2	23.5 24.0 23.4	23.6 23.5 23.7	25.2 25.0 25.0	24.3 24.4 23.9	102.7 ± 1.8	102.9 ± 1.4	1.7	1.4
68.2	71.0 70.9 70.9	77.3 76.7 75.5	71.8 73.4 71.9	71.5 72.2 72.6	77.7 77.9 78.0	74.2 73.3 74.3	104.3 ± 1.5	105.5 ± 2.0	1.4	1.9
113.6	119.7 119.7 119.4	126.6 128.7 129.0	122.6 122.2 125.0	120.7 120.3 118.6	129.7 129.8 130.3	127.0 126.0 127.3	105.6 ± 1.1	106.2 ± 1.5	1.0	1.4

**Table 8:** HPLC method development. HPLC conditions adapted from published HPLC methods for edaravone quantification are appropriately referenced. Other conditions were chosen for reasons outlined in Table 2 in Section 2.

Method	Column	Mobile Phase	Injection Volume (µL)	Flow Rate (mL/min)	Detection Wavelength (nm)	Calibration Standard Solution
1	Agilent ZORBAX Extend-C18 column (250 × 4.6 mm, 5 µm)	Adapted from method of Baghel & Rajput [11] Gradient (time (min)/%B): 0.01/05, 04/15, 10/25, 12/45, 14/25, 16/15, 17/05, 20/05 A: Ammonium acetate buffer (10 mM; pH 5.8) B: 90:10 ACN:MeOH	20 [8,10,11,13]	1 [8,10,13]	243 [8]	Edaravone in ACN [8]
2		Adapted from method of Fanse & Rajput [8] Isocratic: 55% water 45% ACN				
3		Isocratic: 25% water 75% ACN				
4		Gradient (time (min)/B%): 0/25, 7/25, 12/100, 18/100, 20/25, 25/25 A: water B: ACN				
5		Adapted from method of Tanaka et al. [9] Isocratic: 50% MeOH 50% Na_2_HPO_4_ (aqueous; 10 mM; pH 5)				Edaravone in MeOH
6		Adapted from JP method [12] Isocratic: 25% MeOH 75% Acetic acid (0.1% in water; to pH 5.5 with aqueous NH_3_)				
7		Adapted from method of Patel et al. [13] Isocratic: 35% formic acid (0.1% in water) 65% 50:50 ACN:MeOH				
8		Isocratic: 50% formic acid (0.1% in water) 50% 50:50 ACN:MeOH	5			
9	Agilent ZORBAX Extend-C18 column (150 × 4.6 mm, 5 µm)	Isocratic: 65% formic acid (0.1% in water) 35% 50:50 ACN:MeOH				
10		Gradient (time (min)/B%): 0/40, 7/40, 11/50, 16/50, 20/40, 25/40 A: formic acid (0.1% in water) B: 50:50 ACN:MeOH			250	

## Data Availability

The original contributions presented in this study are included in the article. Further inquiries can be directed to the corresponding author(s).
